# PREVALENCE OF AMBIVALENT AGEISM AMONG FAMILY CAREGIVERS AND RELATIONSHIP TO STRESS

**DOI:** 10.1093/geroni/igae098.3257

**Published:** 2024-12-31

**Authors:** Emily Kinkade, Heather Fuller

**Affiliations:** North Dakota State University, Fargo, North Dakota, United States; North Dakota State University, Fargo, North Dakota, United States

## Abstract

Informal family caregivers play an important role in the healthy aging of many older adults. However, little to no research focuses on how family caregivers’ beliefs and attitudes about older adults impact their experience as a caregiver. This study investigates the prevalence of ambivalent ageism - both benevolent and hostile - among informal family caregivers, whether ambivalent ageist beliefs are associated with feelings of stress and burnout, and whether factors such as the relationship quality between the caregiver and care recipient and perceived burden mediate the relationship between ambivalent ageist attitudes and caregiver stress. A sample of informal family caregivers (n=137) was collected from the Midwest. Ambivalent ageist attitudes were measured using the Ambivalent Ageism Scale, family caregiver stress and burden were measured with the Kingston Caregiver Stress Scale and the Zarit Burden Interview respectively, and relationship quality was measured using the Dyadic Relationship Scale. A paired-samples t-test indicated that benevolent ageism was significantly higher than hostile ageism. Multiple regression analyses indicated benevolent ageism was not associated with caregiver stress or burden; however, hostile ageism was significantly associated with caregiver stress and burden. Further, it was found that relationship quality did not mediate the relationship between hostile ageism and caregiver stress, but perceived caregiver burden did mediate this relationship. These findings are discussed and interpreted within the framework of the Informal Caregiving Integrative Model.

